# A Conversation with Marya Lieberman

**DOI:** 10.1021/acscentsci.2c01446

**Published:** 2022-12-13

**Authors:** Dalmeet Singh Chawla

An estimated 10% of medical products in low- and middle-income countries are either falsified or substandard, according to the World Health Organization. It’s particularly difficult in low-income regions to quickly and easily spot subpar medicines and identify their flaws.

**Figure:**
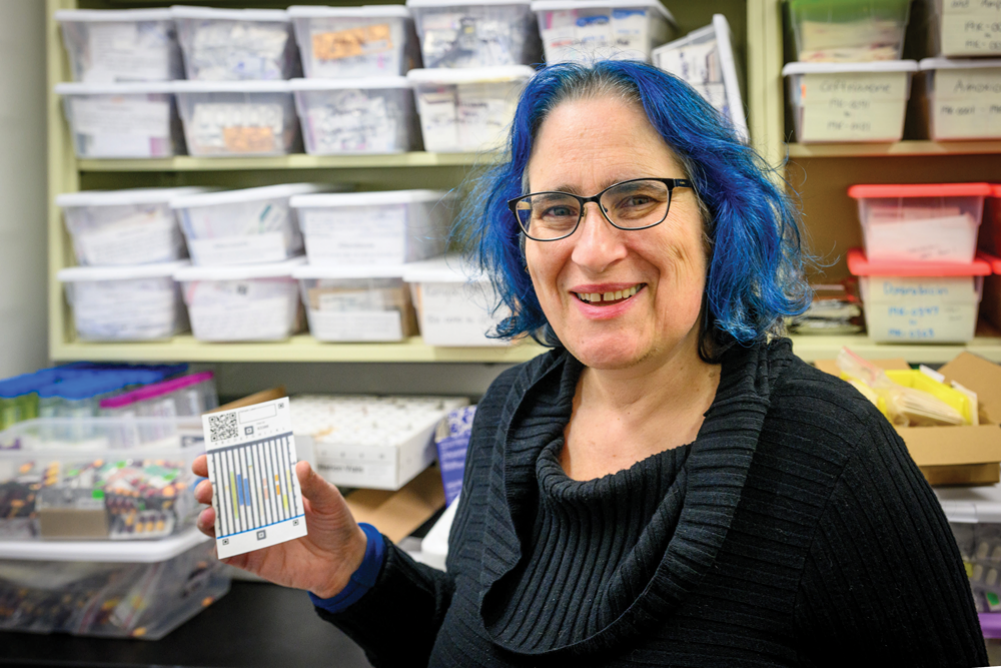
Credit: Matt Cashore/University of Notre Dame

For years, chemist Marya Lieberman of the University of Notre Dame and her team have been developing analytical paper diagnostics that are a cheap, effective, and easy-to-use way of determining whether drug tablets contain the correct medicines. They now want to use the tools they’ve developed to aid harm reduction programs locally and to inform regulators internationally.

Lieberman and her colleagues sell multilane test cards called paper analytical devices (PADs) on their online store. After placing a drug sample on the PAD, users can read the card’s color-based results using an Android application also made by Lieberman’s team.

Dalmeet Singh Chawla spoke with Lieberman about the recent developments in her work and what she hopes to achieve in the future. This interview was edited for length and clarity.

## What problems can PADs solve?

I’m really interested in using color-changing PADs to catch bad-quality medicines. That’s a good application because it’s a low-hanging fruit: pharmaceuticals tend to be high-purity, high-concentration analytes, so analysis of their quality doesn’t require detecting tiny amounts of the target or picking the target out of a complex matrix. Paper diagnostics are not the best for sensitivity and specificity, but another thing to think about is the robustness of the analysis—like if people can get information about their drugs immediately in a field situation. For paper diagnostics, I think we’re often working on analytes where robustness is a really critical component.

We have created a test card for detecting substandard and falsified pharmaceuticals. The PAD detects complete fakes and also has some capacity to detect drugs that don’t contain the right amount of active ingredient. Some lanes will give different colors for different types of pharmaceutical active ingredients (APIs) or different amounts of an active ingredient. So the tests don’t give just a yes-no response.

The 12-lane PAD can analyze more than 60 different pharmaceuticals and is optimized for antibiotics and tuberculosis drugs. All the drugs have different functional groups, which react with the color-changing reagents that are stored in the paper.

**Figure:**
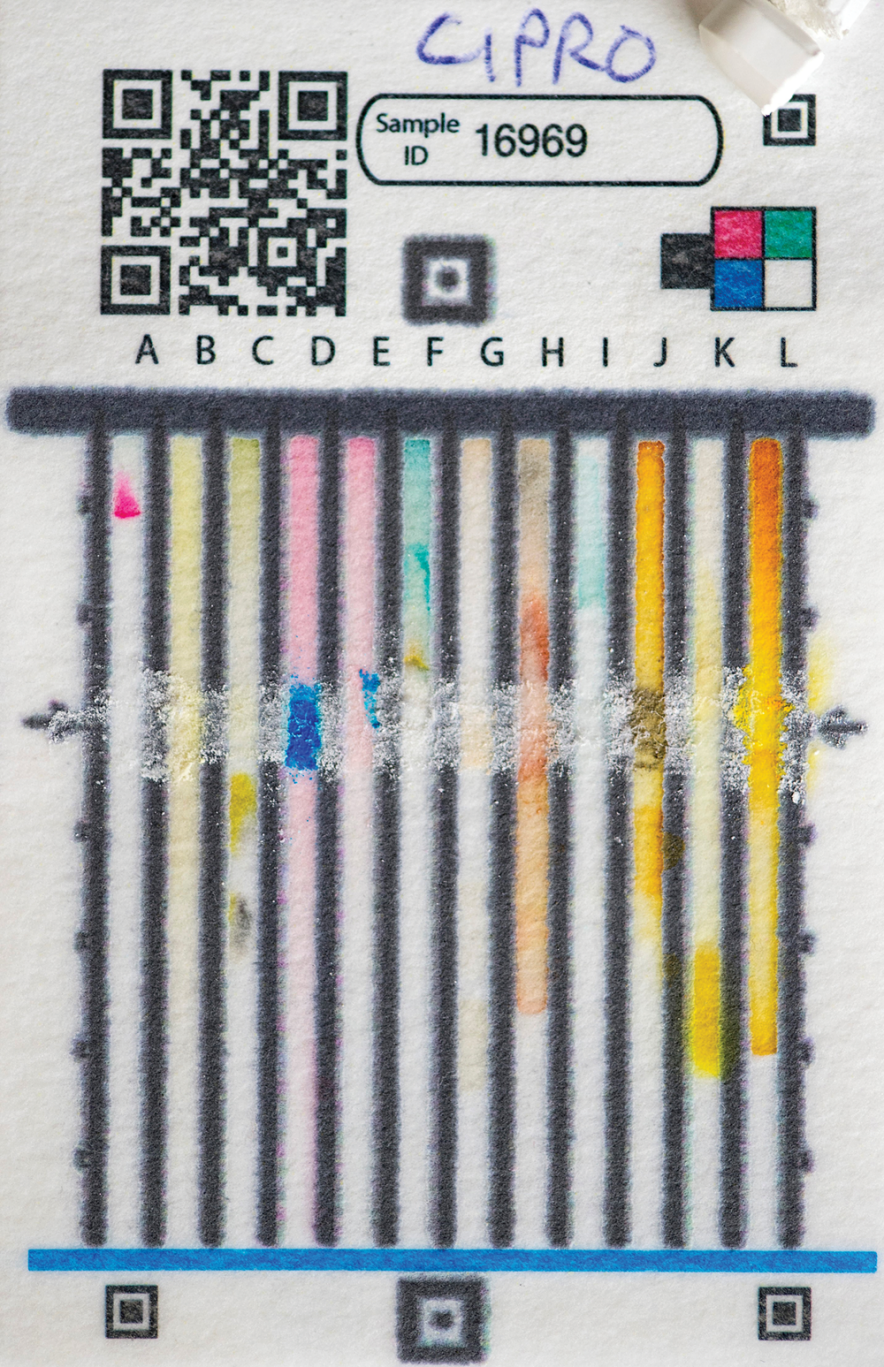
After a sample of the antibiotic amoxicillin is smeared (arrows) across this paper analytical device, water can be drawn up the lanes of the card, creating a multicolor pattern that a smartphone application can read. The application then reports the quality of the sample. Credit: Barbara Johnston/University of Notre Dame.

## Have the tests been rolled out anywhere?

We didn’t reinvent the wheel on this. We’re just using a commercially viable antibody lateral flow assay. We originally developed tests for field screening of illicit drugs, and we now have a test card that we’re using to screen street drugs in a collaboration with harm reduction organizations in Chicago. We’ve also worked with the coroner’s office in Indianapolis.

We’ve been using the tests for pharmaceuticals with partners in Kenya for more than 11 years now. My collaborators at AMPATH, a partnership of U.S. and Kenyan universities and hospitals, are pharmacists. They do covert shopping in a few counties in western Kenya to obtain the medicines for the project, including from medicine shops that aren’t registered. There’s a large informal sector in Kenya.

Then we test out the drugs with the PADs. We keep some samples in Kenya in case the pharmacy board wants to analyze them themselves.

The other samples come to me, and then we analyze some of them by high-performance liquid chromatography to accurately measure the amount of active ingredients in the medicine. I also send samples out to a coalition of about 29 colleges and universities, and they also help analyze samples. These are often undergraduate students who are analyzing samples in their analytical chemistry class or maybe as a research project.

## What have your analyses determined so far?

We’re doing single-tablet analyses to see if the tablet has the correct amount of APIs. What we found so far is that out of 1,100 analyzed pharmaceuticals, 168 failed the tests.

Usually, the quality problem with most of these drugs is that there’s not enough of the API. So if each tablet is supposed to have 100 mg of doxycycline, they’re usually allowed to have between 90% and 110% of that. What they’re not allowed to have is 50 mg of doxycycline and 50 mg of talcum powder, which were the components in one of our samples.

## Is that due to deliberate manipulations of medications?

It’s hard to imagine how else the talcum powder could have come into the medication.

We have identified other instances of manipulation. For example, we have another card called chemoPAD, which we have developed for chemotherapy products. In 2018, one of my students was testing the chemotherapy drug cisplatin being sold at Ethiopian government clinics. We found that the content of cisplatin was systematically underdosed in all those samples and, after more analysis, that there wasn’t the correct amount of the drug in the vial to begin with.

But fraud is not always the source of bad medication. Sometimes a low API is due to natural degradation. For instance, between 2014 and 2016, we were testing out a product from Kenya that is a combination of amoxicillin and clavulanic acid. The tablet is packaged between two pieces of foil that are glued together. That packaging is really important because it protects the amoxicillin and clavulanic acid from oxygen and moisture. We found that there were often pills in the packaging that had undergone some degradation. The clavulanate in most of the cases was not detectable anymore.

That was an example where there was maybe a problem in the packaging of the product, particularly the heat-sealing device that sticks the layers together. We reported the product to the regulatory authority, the Kenyan Pharmacy and Poisons Board, and had some discussion back and forth with the manufacturer.

## What’s the next process after you identify a poor-quality product?

Generally when we find a bad-quality product, I first work with my collaborators who collected the samples, and they reach out to their country’s medical regulatory authority.

In some cases, the authorities want to take some kind of regulatory action before we publish studies about the products. We honor those requests because we don’t want to spoil their ability to respond to a bad-quality product in their country.

## Have any regulatory bodies taken action based on your findings?

Yes, and we’ve seen improvements with the packaging of some products after we’ve reported our problems. But regulatory agencies are very under-resourced and often have a lot on their plate.

Some countries in Africa have shifted from regulatory sampling based on convenience to much more directed, risk-based postmarket surveillance. We have a new project that’s funded by the U.S. National Institutes of Health that’s going to explore integration of the chemoPAD not just into the premarket medical regulatory authority practices in Ethiopia and Kenya but also into clinical practice as a way to protect patients from bad-quality chemo drugs.

*Dalmeet Singh Chawla is a freelance contributor to**Chemical & Engineering News**, the independent news outlet of the American Chemical Society. Center Stage interviews are edited for length and clarity.*

